# Troponina de Alta Sensibilidade em Cirurgia Não Cardíaca

**DOI:** 10.36660/abc.20240312

**Published:** 2024-08-02

**Authors:** Can Özkan

**Affiliations:** 1 Department of Cardiology Bursa City Hospital Bursa Turquia Department of Cardiology, Bursa City Hospital, Bursa – Turquia

**Keywords:** Síndrome Coronariana Aguda, Troponina, Contusões Miocárdicas

**Caro Editor,**

Li com grande interesse o artigo intitulado “Adição de Troponina de Alta sensibilidade à Avaliação de Risco Perioperatório Melhora a Capacidade Preditiva de Morte em Pacientes de Cirurgia não Cardíaca” por Gomes et al.,^1^ a prevalência de lesão miocárdica é maior em pacientes de alto risco, incluindo aqueles com riscos cardiovasculares, cirúrgicos e clínicos. Relataram também que a ocorrência de lesão miocárdica cirúrgica não cardíaca na população não considerada de alto risco não pode ser negligenciada e leva a elevada mortalidade nesta população. No acompanhamento de longo prazo, o prognóstico de pacientes de alto risco sem lesão miocárdica é pior em comparação com pacientes de alto risco sem lesão miocárdica. Alguns comentários podem ser úteis.

As troponinas cardíacas têm sido o biomarcador preferido para identificar lesão miocárdica por um período prolongado.^2^ Com o uso de testes de troponina de alta sensibilidade, a troponina cardíaca pode ser medida em baixas concentrações na maioria dos indivíduos saudáveis e pode estar cronicamente elevada em pacientes com doença cardiovascular estável.^3^ Um estudo descobriu que a incidência de MINS dentro de 30 dias após cirurgia não cardíaca está significativamente associada ao aumento da mortalidade e ao tempo de internação hospitalar. O monitoramento pós-operatório da troponina pode determinar resultados cardiovasculares e facilitar o tratamento precoce.^4^ Acredito que o estudo^1^ é bastante valioso neste aspecto; no entanto, há alguns pontos específicos que gostaria de enfatizar. Em primeiro lugar, a história de infarto do miocárdio prévio foi significativamente maior no grupo MINS. Não está claro no estudo se os níveis elevados de troponina nestes pacientes são secundários à cirurgia não cardíaca ou relacionados à síndrome coronariana aguda. Particularmente, não consegui encontrar informações detalhadas sobre qual porcentagem de pacientes com elevação de troponina superior a 5 vezes o ponto de corte foram submetidos à angiografia coronária ou qual porcentagem exibiu características clínicas de síndrome coronariana aguda.

Neste valioso estudo,^1^ constatou-se que pacientes com alto risco cardiovascular, risco específico da cirurgia ou já considerados de alto risco apresentaram maior prevalência de lesão miocárdica durante cirurgia não cardíaca. Além disso, descobriu-se que a adição de troponina de alta sensibilidade à avaliação de risco aumenta a capacidade de prever a mortalidade por todas as causas a curto e longo prazo. Pode ser um desafio distinguir clinicamente a síndrome coronariana aguda dos níveis elevados de troponina de alta sensibilidade em pacientes no período pós-operatório imediato, e esta não parece ser uma estratégia custo-efetiva. Acredito que os valores de troponina de alta sensibilidade obtidos durante este período poderiam levar a angiografia coronária desnecessária e potencialmente piorar os resultados clínicos. No entanto, parabenizo todos os autores por este estudo muito valioso.
