# Supplementary material for: Troponina de Alta Sensibilidade em Cirurgia Não Cardíaca
Source: Arq Bras Cardiol. 2024 Aug 2;121(7):e20240312. [Article in Portuguese] doi: 10.36660/abc.20240312 (PMC11341205; doi:10.36660/abc.20240312)
Supplement: Translation

# Translation: High-Sensitivity Troponin in Non-Cardiac Surgery

**DOI:** 10.36660/abc.20240312i

**Authors:** Can Özkan

**Affiliations:** 1 Turkey Department of Cardiology, Bursa City Hospital, Bursa – Turkey

**Keywords:** Acute Coronary Syndrome, Troponin, Myocardial Contusions

**Dear Editor,**

I read with great interest the article entitled “Addition of High-Sensitivity Troponin to Perioperative Risk Assessment Improves the Predictive Ability of Death in Non-Cardiac Surgery Patients” by Gomes et al.^1^ the prevalence of myocardial injury is higher in high-risk patients, including those with cardiovascular, surgical, and clinical risks. They also reported that the occurrence of non-cardiac surgical myocardial injury in the population not considered high-risk cannot be overlooked and leads to high mortality in this population. In long-term follow-up, the prognosis of high-risk patients without myocardial injury is worse compared to high-risk patients without myocardial injury. Some comments may be useful.

Cardiac troponins have been the favored biomarker for identifying myocardial injury for an extended period.^2^ With the use of high-sensitivity troponin tests, cardiac troponin can be measured at low concentrations in most healthy individuals and may be chronically elevated in patients with stable cardiovascular disease.^3^ A study found that the incidence of MINS within 30 days after non-cardiac surgery is significantly associated with increased mortality and length of hospital stay. Postoperative troponin monitoring can determine cardiovascular outcomes and facilitate early treatment.^4^ I believe the study^1^ is quite valuable in this regard; however, there are some specific points I would like to emphasize. Firstly, a history of previous myocardial infarction was found to be significantly higher in the MINS group. It is not clear from the study whether the elevated troponin levels in these patients are secondary to non-cardiac surgery or related to acute coronary syndrome. Particularly, I could not find detailed information on what percentage of patients with troponin elevation greater than 5 times the cutoff point underwent coronary angiography or what percentage exhibited clinical features of acute coronary syndrome.

In this valuable study,^1^ it was found that patients at high cardiovascular risk, surgery-specific risk, or those already deemed high-risk, had a higher prevalence of myocardial injury during non-cardiac surgery. Furthermore, the addition of high-sensitivity troponin to risk assessment was found to enhance the ability to predict short and long-term all-cause mortality. It may be challenging to distinguish acute coronary syndrome clinically from elevated high-sensitivity troponin levels in patients in the early postoperative period, and this does not seem to be a cost-effective strategy. I believe that the high-sensitivity troponin values obtained during this period could lead to unnecessary coronary angiography and potentially worsen clinical outcomes. Nevertheless, I congratulate all the authors for this very valuable study.
