# Supplementary material for: Troponina de Alta Sensibilidade em Cirurgia Não Cardíaca
Source: Arq Bras Cardiol. 2024 Aug 2;121(7):e20240312. [Article in Portuguese] doi: 10.36660/abc.20240312 (PMC11341205; doi:10.36660/abc.20240312)
Supplement: Related texts

# Reply: Carta-resposta

**Authors:** Bruno Gomes

**Caro Editor,**

Agradecemos aos autores pelos valiosos comentários em relação ao nosso estudo intitulado “Adição de Troponina de Alta Sensibilidade à Avaliação de Risco Perioperatório Melhora a Capacidade Preditiva de Morte em Pacientes de Cirurgia Não Cardíaca”.^1^

Na verdade, a prevalência de lesão miocárdica relacionada à cirurgia é maior em pacientes com doença arterial coronariana prévia. Contudo, pacientes submetidos a procedimentos cardíacos (por exemplo, cirurgia cardíaca, cateterismo, ablação, etc.) foram excluídos da nossa amostra. Portanto, pacientes que apresentavam características clínicas compatíveis com síndrome coronariana aguda foram excluídos deste estudo. Assim, estamos tratando de dados de um estudo que considerou apenas pacientes com lesão miocárdica após cirurgias não cardíacas (MINS).

O MINS continua sendo uma condição clínica desafiadora, assim como a interpretação das variações de troponina de alta sensibilidade. O reconhecimento da lesão miocárdica tem valor prognóstico e quando não se manifesta em situações típicas de síndrome coronariana aguda (alterações eletrocardiográficas, dor torácica, alterações ecocardiográficas), fica claro que a fisiopatologia não envolve obstrução coronariana aguda e, portanto, há não há necessidade de angiografia coronária.

Portanto, a dosagem rotineira de troponina de alta sensibilidade no pós-operatório agrega valor prognóstico tanto em pacientes de baixo como de alto risco, como demonstrado em nosso estudo. No entanto, ainda temos pouco a oferecer a estes pacientes e devemos concentrar os nossos esforços na redução da mortalidade por esta doença letal.

## References

[1] Gomes BFO, Silva TMBD, Dutra GP, Peres LS, Camisão ND, Homena WS (2024). Addition of High-sensitivity Troponin to Perioperative Risk Assessment Improves the Predictive Ability of Death in Non-cardiac Surgery Patients. Arq Bras Cardiol.

# Reply

**Authors:** Bruno Gomes

**Dear Editor,**

We are grateful to the authors for their valuable comments regarding our study entitled “Addition of High-Sensitivity Troponin to Perioperative Risk Assessment Improves the Predictive Ability of Death in Non-Cardiac Surgery Patients”.^1^

Indeed, the prevalence of surgery-related myocardial injury is higher in patients with previous coronary artery disease. However, patients undergoing cardiac procedures (e.g., cardiac surgery, catheterization, ablation, etc.) were excluded from our sample. Therefore, patients presenting with clinical features consistent with acute coronary syndrome were excluded from this study. Thus, we are dealing with data from a study that considered only patients with myocardial injury after non-cardiac surgeries (MINS).

MINS remains a challenging clinical condition, as is the interpretation of high-sensitivity troponin variations. The recognition of myocardial injury has prognostic value, and when it does not manifest in typical acute coronary syndrome situations (electrocardiographic changes, chest pain, echocardiographic changes), it becomes clear that the pathophysiology does not involve acute coronary obstruction, and therefore, there is no need for coronary angiography.

Therefore, routine high-sensitivity troponin measurement in the postoperative period adds prognostic value in both low and high-risk patients, as demonstrated in our study. However, we still have little to offer to these patients, and we should focus our efforts on reducing mortality from this deadly condition.
